# The associations between intestinal bacteria of *Eospalax cansus* and soil bacteria of its habitat

**DOI:** 10.1186/s12917-022-03223-6

**Published:** 2022-04-02

**Authors:** Yao Zou, Chongxuan Han, Xuxin Zhang, Xiaoning Nan

**Affiliations:** Key Laboratory of National Forestry and Grassland Administration on Management of Western Forest Bio-Disaster, Northwest Agriculture and Forestry University, Yangling, 712100 China

**Keywords:** Zokor, Intestinal bacteria, Environmental bacteria, Low abundance

## Abstract

**Background:**

Intestinal bacteria of mammal can be influenced by many factors, environmental bacteria is an important factor. However, there are few studies on the interactions between environmental bacteria and intestinal bacteria in wild mammals. To explore the associations between the intestinal bacteriome and the related environmental bacteriome, the intestinal bacterial communities of *Eospalax cansus* at three different sites and the bacterial communities of the surrounding soil (outside and inside the cave) at each site were investigated by 16S rRNA sequencing.

**Results:**

The composition and structure between zokor intestinal bacteria and related soil bacteria were distinct, and the soil of zokor habitat harbored significantly higher diversity than that of zokor intestinal bacteria. We have found that host factors may be more important than environmental factors in shaping intestinal bacteriome. In addition, it was found that the relative abundances of shared OTUs between zokors and related soil were significantly negatively related. These shared OTUs were present in the soil at relatively low abundance. However, these shared OTUs between zokors and soil were affiliated with diverse bacterial taxa, and they were related to the degradation of complex carbohydrates.

**Conclusions:**

These results suggested that the zokor gut may mainly select for low-abundance but diverse soil bacteria, which may be a host- specific choice for zokor to meet the needs of its phytophagous dietary.

**Supplementary Information:**

The online version contains supplementary material available at 10.1186/s12917-022-03223-6.

## Background

The genomes of intestinal microbes in mammals contain more than 100 times as many genes as the host genomes [1]. Intestinal microbes of mammals play a crucial role in physiological functions, such as the digestion and absorption of food, energy acquisition [2, 3], nutrition regulation and immune response of host [4–6]. The intestinal microbes are also associated with the body balance and health status of host, they can be regarded as an “organ” playing an important part in the metabolic process [7–9].

Zokor is one of subterranean rodents endemic to east Asia [10]. It is a small herbivorous mammal who gnaw the roots of plants for food and could cause large-scale disasters to woodland and meadow [11]. Studies of zokor have focused on the classification and phylogeography [12–16], while the research on microecology of zokor are scarce. Previous studies indicated that the season and diet had effect on intestinal microbes of zokor based on previous studies [17–19]. However, it is unclear whether there are other factors have effects on shaping zokor intestinal microbes.

Intestinal microbes of mammal are influenced by host and environment factors [20–22]. Host factors include genetic background, age, gender and the health status of host [23]. Environment include many factors, such as food [24, 25], season [26, 27], environment microbes and geographical location. Environment microbes especially soil microbes were important for wild animals in grassland ecosystem. On the one hand, microbes in the soil could drive the transformation and recycling of organics and nutrition, higher diversity of soil would be beneficial to improve the soil fertility and thus affecting the health condition of animals [27, 28]. On the other hand, the activities of humans and animals would affect the diversity of soil microbes [29]. Recent studies have found that the digging activities of zokors have altered soil nutrients and plant communities [30, 31]. However, few studies have directly estimated the relationship between the intestinal bacteriome of wild animals and the related environmental bacteriome. The interaction between zokor intestinal microbiota and soil microbiota is not well understood.

To understand the associations between the zokor intestinal bacteriome and the related environmental bacteriome, we have investigated the composition and structure of intestinal bacterial communities of *Eospalax cansus* at three different sites and related soil bacterial communities (outside and inside the cave) at each site based on 16S rRNA sequences. In addition, we explored the possible associations between the zokor intestinal bacteriome and the related environmental bacteriome. This work would be significant to improving understanding of interactions between zokor intestinal bacteria of and related soil bacteria in wild environments.

## Results

### Comparison of composition and structure between zokor intestinal bacteriome and soil bacteriome

**Fig. 1:**
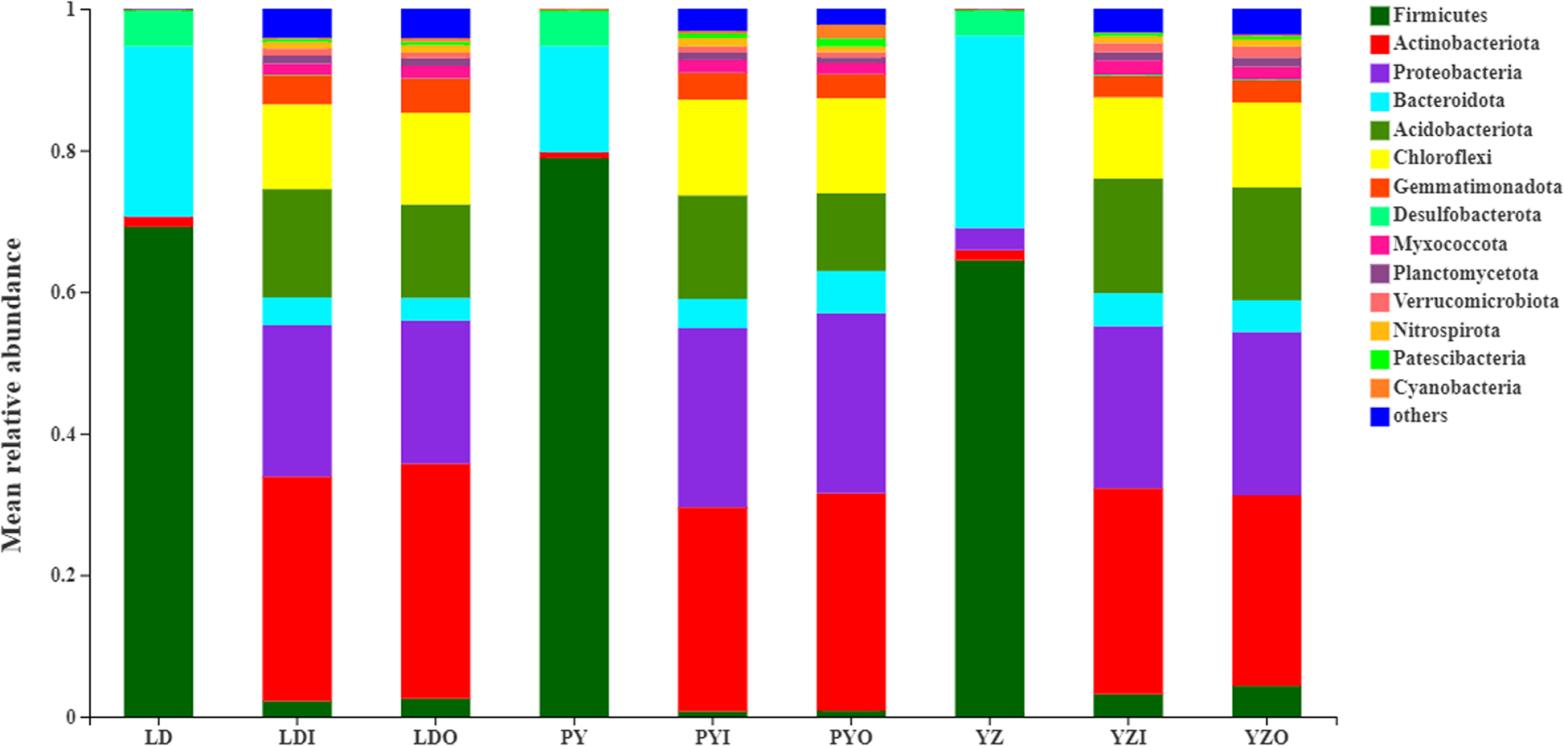
Mean relative abundances of bacterial phyla across zokors (LD, zokors from LD; PY, zokors from PY; YZ, zokors from YZ) and soil samples (LDI: soil inside the cave from LD; LDO: soil outside the cave from LD; PYI: soil inside the cave from PY; PYO: soil outside the cave from PY; YZI: soil inside the cave from YZ; YZO: soil outside the cave from YZ) at three different sites. Only those phyla with > 0.01% mean relative abundance across all samples are shown

At phylum level, the zokor intestinal bacteriome across all sites were dominated by Firmicutes and Bacteroidota, followed by Desulfobacterota and Proteobacteria (> 1% relative abundance), with mean relative abundances across all zokor samples of 70.11, 22.69, 4.32, and 1.30%, respectively. However, the bacteriome of soil outside the cave were dominated by Actinobacteria (30.29%), followed by Proteobacteria (22.90%), Acidobacteria (13.37%) and Chloroflex (12.79%). In contrast, the bacteriome of soil inside the cave mainly consisted of Actinobacteria (29.88%), followed by Proteobacteria (23.20%), Acidobacteria (15.37%) and Chloroflex (12.32%). The mean relative abundance of intestinal bacteriome of zokor, soil bacteriome (outside or inside the cave) at phylum level in each site was shown in Fig. 1. At genus level, the composition and structure between zokor intestinal bacteria and related soil bacteria were also largely distinct (Fig. S1).

**Fig. 2:**
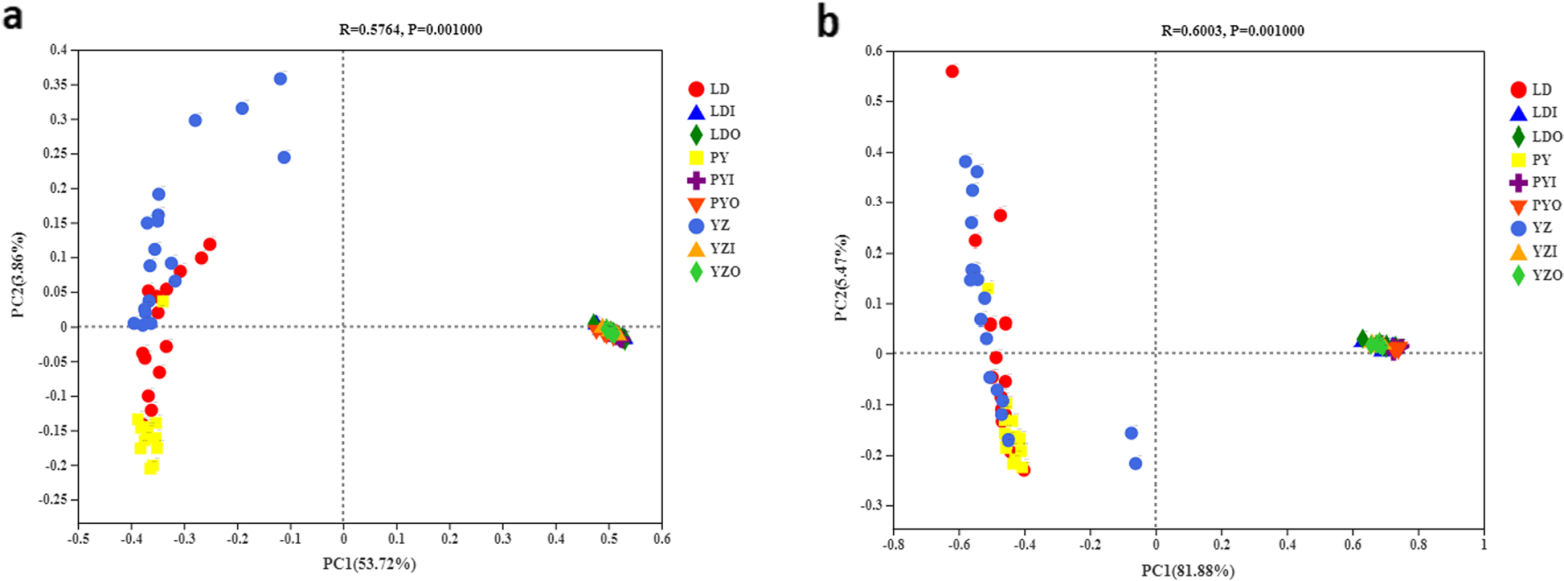
Principal coordinates analysis (PCoA) of zokor and soil bacterial communities across 75 samples based on (**a**) the Bray-Curtis distance metrics. (**b**) the weighted UniFrac distance metrics. Key: zokor samples (LD, zokors from LD; PY, zokors from PY; YZ, zokors from YZ), soil samples (LDI: soil inside the cave from LD; LDO: soil outside the cave from LD; PYI: soil inside the cave from PY; PYO: soil outside the cave from PY; YZI: soil inside the cave from YZ; YZO: soil outside the cave from YZ)

Alpha diversity of zokor and soil (outside the cave and inside the cave) bacterial communities in each site were investigated. The diversity of intestinal bacteriome of zokors were lower than those of soil, while there were no differences between soil outside the cave and that inside the cave (Fig. S2). Differences between the zokor and soil bacterial community structure were evident based on the Bray-Curtis (ANOSIM, *r* = 0.5764, *P* < 0.001; Fig. 2a) and weighted UniFrac distance metrics (ANOSIM, *r* = 0.6003, *P* < 0.001; Fig. 2b). The bacteriome of the soil outside the cave and the soil inside the cave were more similar to each other than the zokor intestinal bacteriome. Despite a partial overlap, the zokor had distinct bacterial communities among sampling sites based on Bray-Curtis distance metrics (ANOSIM, *r* = 0.2458, *P* < 0.001; Fig. S3).

### Microbes that were abundant in zokor guts were present in the soil at relatively low abundance

**Fig. 3:**
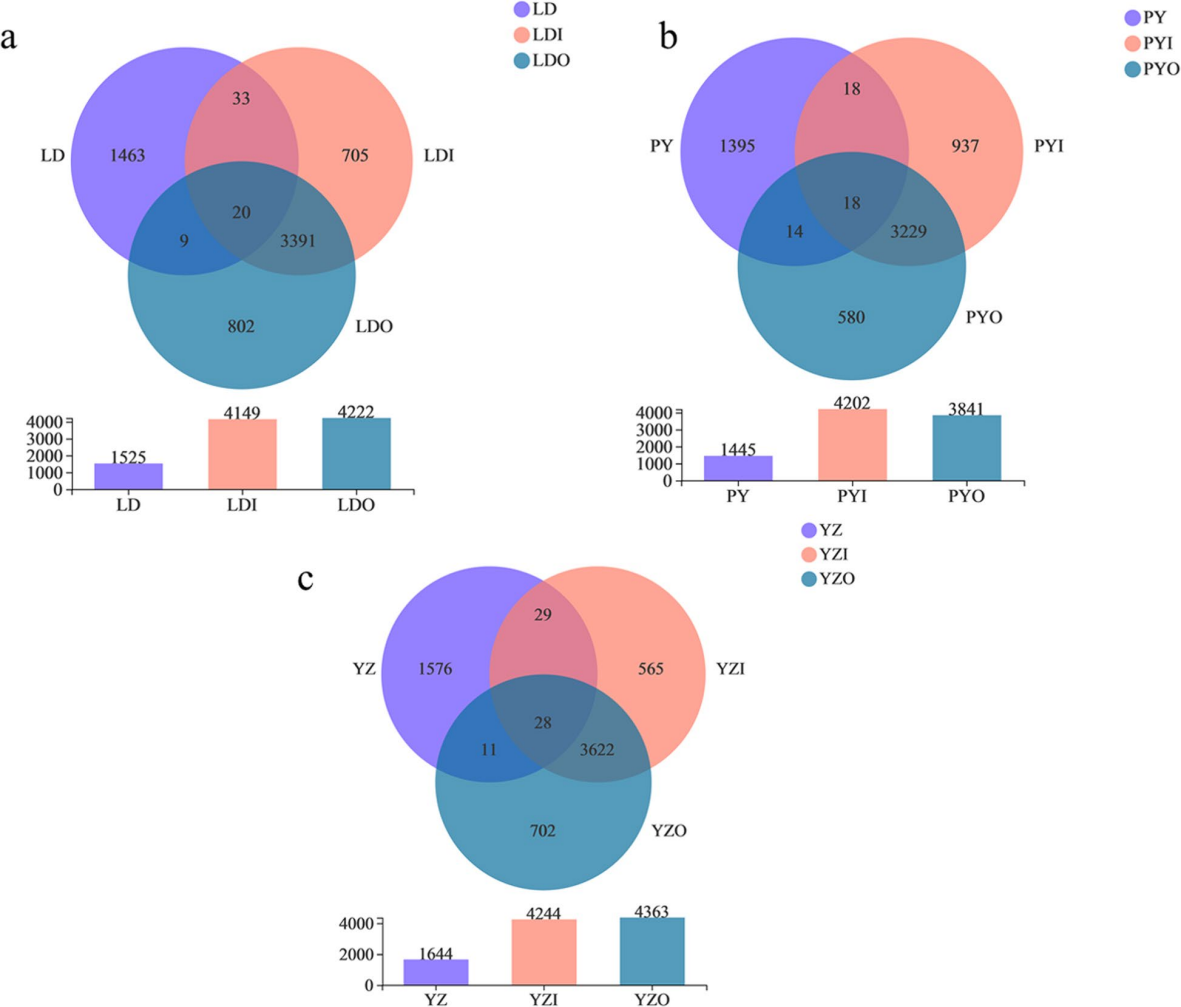
Venn diagram showing the shared and unique OTUs among zokor and soil bacteriome in each site. (**a**) samples from LD. (**b**) samples from PY. (**c**) samples from YZ

We calculated the shared and unique OTUs among zokor and soil bacteriome in each site (Fig. 3). Most OTUs in zokor guts were not observed in the environmental samples (Fig. 3; Table 1). The proportion of unique gut OTUs in zokors in LD, PY and YZ were 95.93% (1463 of 1525 total OTUs), 96.54% (1395 of 1445 total OTUs) and 95.86% (1576 of 1644 total OTUs), respectively. In particular, the proportion of shared OTUs between zokor and the soil outside the cave were 1.90, 2.21, and 2.37% at those three sampling sites of LD, PY and YZ, respectively, whereas the corresponding proportion of shared OTUs between zokor and the soil inside the cave were 3.48, 2.49 and 3.47%, respectively. There was a large-scale overlap between the soil microbial communities that outside the cave and inside the cave (Fig. 3). Furthermore, 1248.

**Table 1:** Venn diagram summarizing the overlap of soil (inside and outside) and *Eospalax cansus* OTUs at different sites

Location	LD	PY	YZ
Total OTUs	1525	1445	1644
Shared gut-soil (outside) OTUs	1.90%	2.21%	2.37%
Shared gut-soil (inside) OTUs	3.48%	2.49%	3.47%
Shared gut-environment OTUs	4.07%	3.46%	4.14%
Unique gut OTUs	95.93%	96.54%	95.86%
Shared gut OTUs in different sites	81.84%	86.37%	75.91%

OTUs were shared among zokors of the three sites (Fig. S4). The proportion of shared OTUs among zokors at three sites were 82, 86, and 76% in LD, PY and YZ, respectively.

Most OTUs (> 90%) that were shared between zokor and the soil were at relative abundances of 0.5% or less in the soil (Fig. S5). Using Spearmen’s correlation tests, it was found that the relative abundances of shared OTUs between zokors and related soil were significantly negatively related (Fig. S5a, *p* < 0.01; Fig. S5b, *p* < 0.01; Fig. S5c, *p* < 0.01). Those OTUs that were abundant in zokor guts showed a relatively low abundance in the soil (outside or inside the cave), And the more abundant soil OTUs had a relatively low abundance in zokor gut regardless of sites.

The core zokor gut bacterial communities were defined as those OTUs that were present on at least 90% of all individuals in each zokor species. The core bacterial communities of zokor included 311 OTUs (Table S1). The taxonomic status (at family level) and mean relative abundances of these OTUs were listed in Table 2. It was clearly that the abundances of core OTUs were high in zokor gut, while these OTUs had a relatively low abundance in the soils (Table 2). In addition, the majority of the zokor core microbes were enriched in bacterial taxa that were not observed in soil samples. The minority of the core OTUs (13, 14 and 4 of 311 in the soil outside the cave, and 31, 18 and 18 of 311 in the soil inside the cave in LD, PY and YZ, respectively) were only sporadically observed in the soil, and they had low abundances (< 0.1%) in soil samples. We also calculated 84 most abundant OTUs in soil (> 0.1% relative abundance) (Table S2), however, most of the most abundant soil OTUs (73 of 84 OTUs) were not present in zokor guts. The rest of most abundant OTUs in soil were present in zokor gut at < 0.1% relative abundance. Furthermore, there was no overlap occurred in the zokor core bacteria (311 OTUs) and the most abundant soil bacteria (84 OTUs).

**Table 2:** The mean relative abundances of *E. cansus* core OTUs (***≥***90% prevalence in all samples) in zokor guts and in the environments at three different sites

		LD	LDI	LDO	PY	PYI	PYO	YZ	YZI	YZO
Family	NO									
Anaerovoracaceae	3	0.61	0.00	0.00	0.48	0.00	0.00	0.77	0.00	0.00
Butyricicoccaceae	1	0.11	0.00	0.00	0.05	0.00	0.00	0.15	0.00	0.00
Christensenellaceae	6	1.41	0.00	0.00	1.11	0.00	0.00	0.83	0.00	0.00
Coriobacteriales_ Incertae_Sedis	1	0.02	0.00	0.00	0.02	0.00	0.00	0.27	0.00	0.00
Desulfovibrionaceae	50	3.81	0.00	0.00	3.93	0.00	0.00	2.56	0.00	0.00
Eggerthellaceae	6	0.00	0.00	0.00	0.24	0.00	0.00	0.00	0.00	0.00
Lachnospiraceae	96	0.23	0.01	0.00	0.27	0.00	0.00	0.25	0.00	0.00
Monoglobaceae	2	26.95	0.03	0.01	31.15	0.02	0.01	22.82	0.01	0.00
Muribaculaceae	50	0.19	0.00	0.00	0.15	0.00	0.00	0.00	0.00	0.00
norank_o_Clostridia	1	0.18	0.00	0.00	0.15	0.00	0.00	0.20	0.00	0.00
norank_o_Clostridia_UCG-014	6	12.19	0.00	0.00	9.20	0.00	0.00	15.78	0.00	0.00
norank_o_Clostridia_vadinBB60_group	10	0.03	0.00	0.00	0.04	0.00	0.00	0.00	0.00	0.00
norank_o_Coriobacteriales	1	0.11	0.00	0.00	0.17	0.00	0.00	0.04	0.00	0.00
norank_o_RF39	1	0.33	0.00	0.00	0.31	0.00	0.00	0.26	0.00	0.00
Oscillospiraceae	49	0.84	0.00	0.00	1.00	0.00	0.00	0.49	0.00	0.00
Peptococcaceae	2	0.14	0.00	0.00	0.08	0.00	0.00	0.38	0.00	0.00
Rikenellaceae	1	0.03	0.00	0.00	0.02	0.00	0.00	0.02	0.00	0.00
Ruminococcaceae	19	14.03	0.00	0.00	13.78	0.00	0.00	12.06	0.00	0.00
Saccharimonadaceae	2	0.09	0.00	0.00	0.20	0.00	0.00	0.00	0.00	0.00
UCG-010	4	0.19	0.00	0.00	0.19	0.00	0.00	0.22	0.00	0.00
unclassified_o_ Coriobacteriales	1	0.07	0.00	0.00	0.15	0.00	0.00	0.05	0.00	0.00

**Fig. 4:**
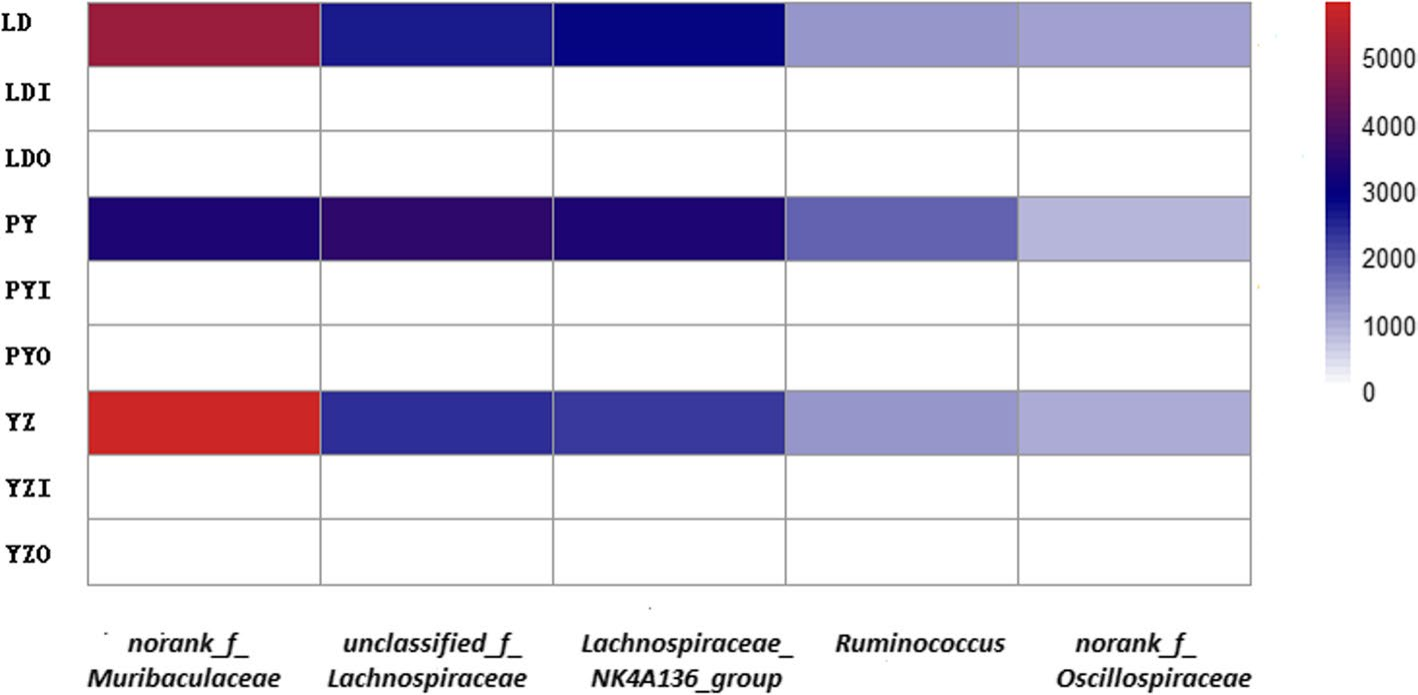
The five dominant genera (> 1% meanrelative abundance) across all zokor samples (LD, zokors from LD; PY, zokors from PY; YZ, zokors from YZ) and corresponding abundance across soil samples (LDI: soil inside the cave from LD; LDO: soil outside the cave from LD; PYI: soil inside the cave from PY; PYO: soil outside the cave from PY; YZI: soil inside the cave from YZ; YZO: soil outside the cave from YZ) in each site

At genus level, the abundance of five most dominant genera in zokor guts were calculated, including *norank_f_Muribaculaceae, unclassified_f_Lachnospiraceae, Lachnospiraceae_NK4A136_group, Ruminococcus*, and *norank_f_Oscillospira* with mean relative abundance across all zokor samples of 21, 13, 12, 6 and 4%, respectively. However, the mean abundances of these five genera in soil bacteriome (outside or inside the cave) were all lower than 0.01%. The mean relative abundances of these five genera across all zokor samples and related soil samples in each site were shown in Fig. 4.

### The shared OTUs between zokor and soil represent diverse microbial taxa

We calculated the taxonomic composition and the mean relative abundance of shared OTUs between zokor and soil. Although the proportion of shared OTUs between zokor and the soil were less than 5% at each site (Table 1), those shared OTUs represent diverse microbial taxa including 13 phyla and 97 genera, respectively. Despite a little difference across sites, the major phyla (> 1% average relative abundance) of these shared OTUs included Firmicutes and Bacteroidota, followed by Actinobacteriota, Proteobacteria and Desulfobacterota (Fig. S6). These phyla were similar to the major composition of intestinal microbes of zokors (Fig. 1). At genus level (Fig. S7), *norank_f_Muribaculaceae*, *unclassified_f_Lachnospiraceae*, *Lachnospiraceae_NK4A136_group*, *norank_f_Lachnospiraceae*, Ruminococcus and *Lactobacillus* (> 1% average relative abundance) were dominant based on the taxonomic composition of the shared OTUs. These major genera of shared OTUs were also the main genera of intestinal bacteria of zokors instead of soil bacteria of its habitat (Fig. S7, Fig. S1). The composition of shared OTUs were similar to that of the zokor’s intestinal microbes at both phylum and genus level.

### Predicted metagenomes

**Fig. 5:**
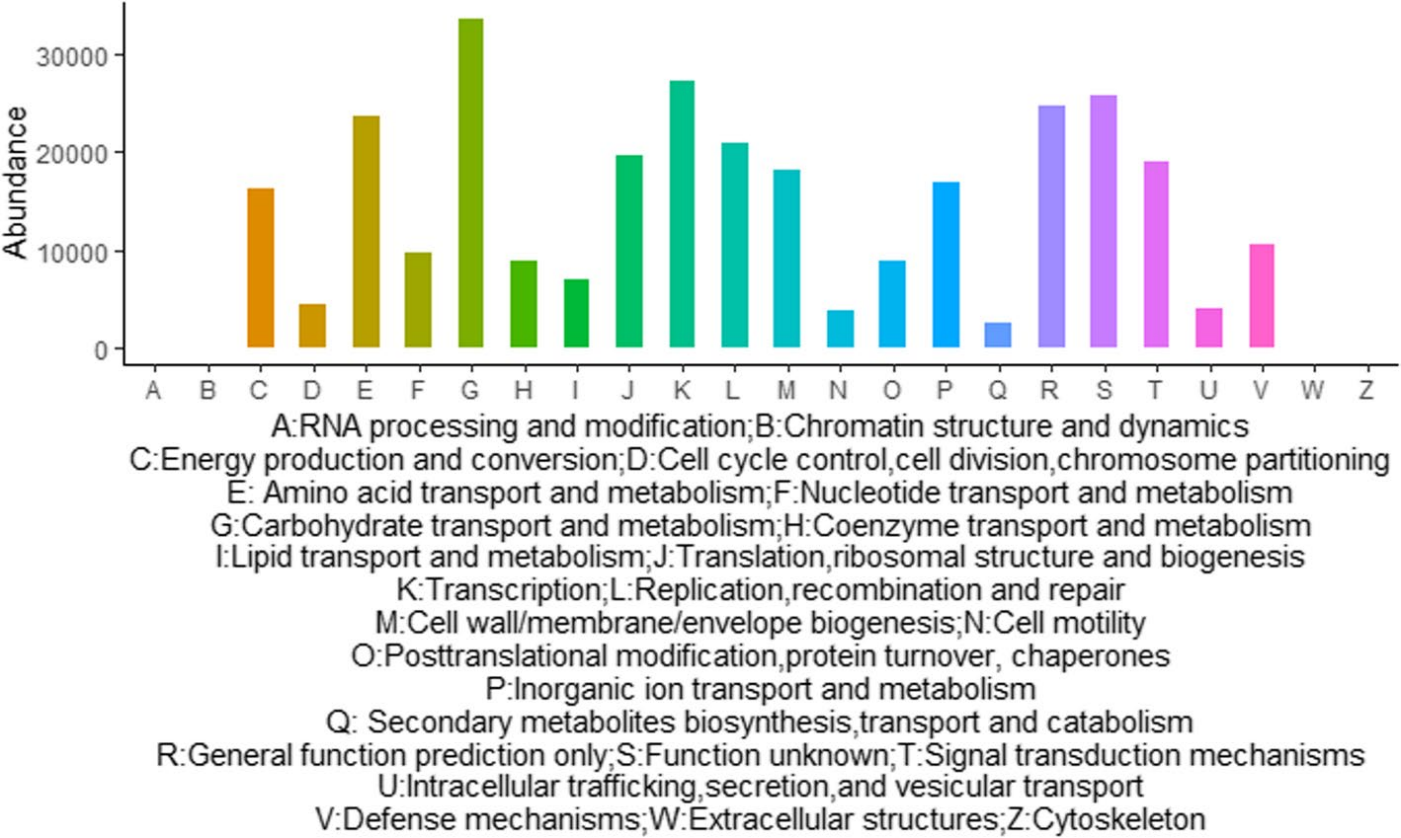
The abundance presented in zokor of functional genes of shared OTUs between zokor samples and soil samples based on COG database

The function of microbes of zokor and soil based on COGs include 25 functions (Table S3). The relative abundances of all categories of function genes were highly significantly higher in soil than those in *E. cansus* (*p* < 0.01). Amino acid transport and metabolism, general function prediction only, and energy production and conversion were the most important functions for soil microbes, while carbohydrate transport and metabolism, transcription, general function prediction only, and amino acid transport and metabolism were the most important functions for intestinal microbes of zokors. In addition, we found 25 gene functions were present in shared OTUs (between zokor and soil) based on COG database (Fig. 5). We also found that carbohydrate transport and metabolism, transcription, general function prediction only, and amino acid transport and metabolism were the most important functions of shared OTUs.

## Discussion

### The differences of composition and diversity between zokor intestinal bacteriome and environmental bacteriome

In this study, the composition and structure of intestinal microbes of *E. cansus* and soil microbes of its habitat were studied by high-throughput sequencing technology. At phylum level, Firmicutes and Bacteroidota are dominant microbes in zokor (Fig. 1). The total proportion of Firmicutes and Bacteroidota accounted for more than 90% of 16S rRNA sequences. Firmicutes and Bacteroidota are mainly responsible for food fermentation in the gut [32]. Some herbivores, such as horse, donkey and rabbits, usually harbored high percentage of Firmicutes and Bacteroidota [33–35]. High percentage of Firmicutes and Bacteroidota contributes to decomposing the cellulose and hemicellulose. Therefore, the composition of intestinal microbes of *E. cansus* indicates that intestinal microbes of zokor are highly adaptive to phytophagous habits. However, Actinobacteria and Proteobacteria were two dominant phyla in soil (outside the cave or inside the cave), and the proportion of these two phyla could account for more than 50% of 16S rRNA sequences (Fig. 1). It is clearly that the composition of zokor intestinal microbes and soil microbes of its habitat were largely distinct.

Soil bacteriome harbors more OTUs and higher alpha diversity than that of zokor intestinal bacteriome (Fig. 3; Fig. S2). It was indicated that the soil bacteria may have more physiological functions, such as decomposing the organics, transforming nutrition, decontaminating pollutant, and involved in metabolism and cycles of elements [36, 37]. This is further supported by the fact that the abundances of all categories of functional genes were highly significantly higher in soil than those in *E. cansus* based on predicted metagenome. It is implicated that soil has a strong metabolic ability, and it could decompose multiple substances such as amino acid, coenzyme, lipid and carbohydrate.

### Host factors may be more important than environmental factors in shaping intestinal bacteriome

We have found that the composition and structure of zokor intestinal bacteriome among three sites were more similar to each other than that between zokor intestinal bacteriome and soil bacteriome, and soil bacteriome were also gathered together regardless of inside or outside the cave in PCoA analysis (Fig. 2). It was suggested that host factors were more important than environmental factors in shaping intestinal bacteriome. This result was also supported by the fact that the proportion of shared OTUs among zokors of three sites was much higher than that between zokor and related soil. Host factors appear to select for and maintain the intestinal bacteriome at similar composition and structure regardless of habitat [38]. The same patterns have been found in pikas [38], amphibians [23, 39, 40] and humpback whales [41].

Host genetic factors may have a stronger effect on core bacteria of zokor than that of environmental factors. We have found that all of dominant soil OTUs were not the members of the zokor core bacteria, probably based on the flow of non-resident, transient bacteria associated with ingested food [38]. In addition, although the composition of core OTUs of zokor is diverse, most of core OTUs in zokors were not detected in the soil at all. Therefore, these core microbes may be transmitted vertically from parents, or horizontally by conspecifics. However, zokor live alone across its whole life except for breeding seasons [42]. They hardly have social behavior so that the transmission of microbes among individuals was limited. Thus, the investigation of the vertical transmission of zokor from parents to offspring was needed to explain these findings in future study.

### Zokor gut may select for rare but diverse soil bacteriome to meet the needs of its phytophagous dietary

Zokor gut may select for rare but diverse soil bacteriome (outside or inside the cave). The relative abundances of shared OTUs between zokors and soil were negatively related (Fig. S5). Those shared OTUs that were abundant in zokor guts showed a relatively low abundance (< 0.5%) in the soil, but they represent diverse microbial taxa**.** In addition, the five predominant genera in zokor gut (*norank_f_Muribaculaceae*, *unclassified_f_Lachnospiraceae*, *Lachnospiraceae_NK4A136_group*, *Ruminococcus*, *norank_f_Oscillospira*) were also rare (< 0.01%) in soil bacteriome (outside or inside the cave). It is indicated that zokor gut may select for low abundance soil bacteriome. The same pattern has been observed in pika [38], amphibian [39], and crustacean [43] systems.

Shared OTUs between zokor and soil were affiliated with diverse bacterial taxa, and they were related to the degradation of complex carbohydrates. At phylum level, high percentage of Firmicutes and Bacteroidota contributes to decomposing the cellulose and hemicellulose [32]. At genus level, *norank_f_Muribaculaceae* might be related to the degradation of a variety of carbohydrates [44]. *Unclassified_f_Lachnospiraceae* and *Lachnospiraceae_NK4A136_group* both belong to Lachnospiraceae which are involved in metabolism as butyrate producer [45, 46]. And the latter was fibrolytic bacterium which can degrade the complex plant bran of recalcitrant substrate [47, 48]. *Lactobacillus* could ferment the carbohydrates to lactic acid [49]. The composition of shared OTUs were related to the degradation of complex carbohydrates. This result was also supported by the fact that the most important function of shared OTUs (between zokor and soil) were related to carbohydrate transport and metabolism (Fig. 5), which were also the most important function of intestinal microbes of zokor (Table S3). The interactions between the intestinal microbes of zokor and soil microbes of its habitat indicated its high adaptation of phytophagous habits. To meet the needs of zokor’s phytophagous dietary, zokor gut may select for environment microbes with specific functions such as carbohydrate degradation, which may be a host-specific choice of zokor.

## Conclusions

In conclusion, our study demonstrates that the composition and structure between the intestinal microbes of *Eospalax cansus* and soil microbes of its habitat are largely distinct. We also found that host factors may be more important than environmental factors in shaping intestinal bacteriome. In addition, the shared OTUs between zokors and related soil were present in the soil at relatively low abundance. However, those shared OTUs were affiliated with diverse bacterial taxa, and they were related to the degradation of complex carbohydrates. These results suggested that the zokor gut may mainly select for low-abundance but diverse soil bacteria, which may be a host- specific choice for zokor to meet the needs of its phytophagous dietary.

## Methods

### Sample collection

Zokor samples (*E. cansus*) used in this study were collected from three sites of Ningxia Hui Autonomous Region between May and July 2020. Zokors were humanely euthanized by intravenous pentobarbital sodium (390 mg/mL) overdose after sedation with xylazine hydrochloride (5 mg/kg) [19]. The cecal contents of zokors were collected in cryopreservation tubes within 5 min, immediately stored in liquid nitrogen. A total of 45 cecal samples were obtained from *E. cansus* at three sites. Experiments were approved by the Institution of Animal Care. Sample collection process of wild zokors follows the guidelines of our academic institution.

**Fig. 6:**
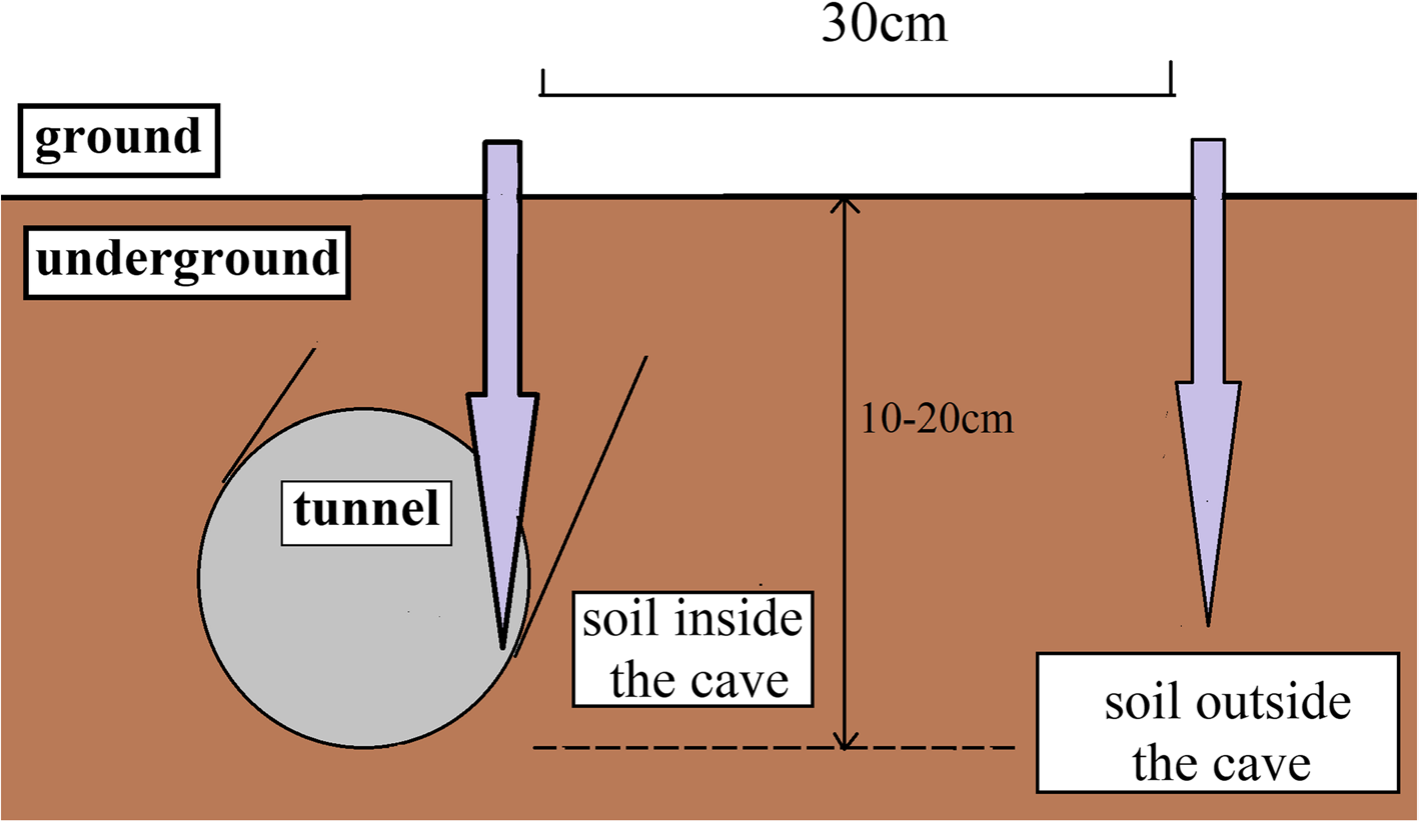
The schematic drawing showing the location of soil samples (outside or inside the cave) within each plot

To understand the environmental bacteriome of the zokors’ habitats, we collected 30 soil samples (10–20 cm; 10 samples per site, including 5 soil samples outside the cave and 5 soil samples inside the cave) from the three sites. Within each site, 5 plots (1 × 1 m^2^) were randomly placed, with the stipulation that the plots were at least 10 m apart. Within each plot, each sample was a mixture of 5 individual soil cores at the depth of 10–20 cm. The schematic drawing showing the location of soil samples (outside or inside the cave) within each plot was shown in Fig. 6. All the soil samples were transported to our laboratory, and stored at − 80 °C for bacterial community analysis. The details of sampling information were shown in Table 3.

**Table 3:** Information of sampling area of *E. cansus*

Sampling locality	Code	Longitude/°E	Latitude /°N	Altitude/m	Gut sample size (male/female)	Soil samples size (outside/inside)
Shenlin forest in Longde county	LD	105.9290	35.579	1824	14 (8/6)	10 (5/5)
Xiaoshigou village in Pengyang county	PY	106.8456	36.0273	1721	13 (3/10)	10 (5/5)
Hongzhuang forest in Yuanzhou	YZ	106.1143	35.8121	2202	18 (12/6)	10 (5/5)

The major plant community in each sampling site was identified based on morphological characteristics. The plant community in LD were dominated by *Corylus heterophylla*, *Hemerocallis citrina* and *Isatis tinctorial*. *Malus pumila* and *Isatis tinctorial* were dominant plants in PY, and *Larix gmelinii*, *Amygdalus davidiana*, *Hippophae rhamnoides* and *Urtica fissa* were dominant plants in YZ.

### DNA extraction

Total genomic DNA of cecal contents and soil were extracted with a Stool Genome DNA Extraction Kit (Tiangen Inc.) and E. Z. N. A. @soil DNA Kit (Omega Bio-tek, Norcross, GA, U.S.) following the manufacturer’s protocol, respectively. DNA concentration and quality were determined using the Nanodrop 2000 Spectrophotometer. DNA were detected with 1% agarose gel extraction kit (Takara Inc.) and then purified and sequenced by Majorbio Bio-Pharm Technology Co. Ltd. (Shanghai, China).

### PCR amplification and MiSeq sequencing of 16S rRNA gene

The universal primer pair (338 F: 5′-ACTCCTACGGGAGGCAGCAG-3′, 806 R: 5′-GGACTACHVGGGTWTCTAAT −3′) was used to amplify the 16S rRNA gene (V3-V4 hypervariable regions) from cecal contents and related soil DNAs [26]. The procedures of PCR amplification, agarose gel extraction and further purification were described in previous study [19].

Sequencing of 16S rRNA were performed using an Illumina MiSeq platform (Illumina, San Diego, USA) according to the protocols by Majorbio Bio-Pharm Technology Co. Ltd. (Shanghai, China).

### Processing of sequencing data

Raw fastq files were quality-filtered by Trimmomatic and merged by FLASH (version 1.2.11 https://ccb.jhu.edu/software/FLASH/index.shtml). The criteria of quality control refer to previous study [19].

Sequences were clustered into operational taxonomic units (OTUs) at 97% identity threshold using UPARSE (version 7.1 http://drive5.com/uparse/) [50]. The taxonomy of each sequence was annotated and identified by RDP Classifier (version 2.1.1 https://sourceforge.net/projects/rdp-classifier/) based on Silva (SSU123) 16S rRNA database [38].

### Bioinformatics analysis

QIIME Pipeline Version 1.9.1 [51] was used to analyze raw data. All reads were trimmed and then assigned to each sample based on their unique barcodes. After removing chimeras, all the reads were clustered into operational taxonomic units (OTUs) at a 97% sequence identity, and were identified at different level of classification [50, 52]. To standardize sampling efforts across samples, each sample was rarefied to the same number of reads (22,494 sequences). The rarefaction curves were generated based on the observed OTUs [53]. Thereafter, the mean relative abundances of OTUs were calculated for each sample. The core microbes were defined as those OTUs that are present on at least 90% of all zokor samples. Shannon [54] and Chao [55] indices of intestinal microbiota and soil microbiota were calculated by QIIME to evaluate the alpha diversity. To assess beta diversity, principal coordinate analysis (PCoA) was performed based on Bray-Curtis and unweighted UniFrac distance to visualize the separation of intestinal microbiota and soil microbiota structure across different sites [56, 57].

### Statistical analysis

Statistical analyses were conducted through SPSS 23.0 software [58, 59]. The significant and the highly significant levels were 0.05 and 0.01, respectively. The differences between intestinal microbes and soil microbes were detected by Wilcoxon rank sum test [60].

Venn diagrams were drawn in RStudio (version 1.3.1093.0) to visualize the shared OTUs between zokor intestinal microbes and related soil microbes in each site. At OTU level, the proportion of zokor intestinal microbes that were also in the soil in each site were calculated. In addition, we calculated the mean relative abundances and total abundance of shared OTUs between zokor intestinal microbes and related soil microbes. The composition and proportion of these shared OTUs were calculated at phylum and genus level. Pie charts were created to visualize the composition and relative abundance of shared OTUs between zokor intestinal microbes and related soil microbes at phylum and genus level in each site.

### Predicted metagenomes

PICRUSTv1.1.0 [61] was used to predict the function based on the abundance of COGs. By comparing the 16S rRNA gene sequenced with the reference genome database of microorganisms with known functions, the function can be predicted. We predicted the metagenome for zokor OTUs and for the soil OTUs (outside or inside the cave). In addition, we predicted the metagenome of shared OTUs (between zokor and soil) based on the abundance of shared OTUs presented in zokor. Two-tailed t tests (Bonferroni corrected) were performed to test the differences of gene functions between intestinal microbes of *E. cansus* and soil microbes of its habitats.

### Nucleotide sequence accession numbers

The raw data of 16S rRNA sequence were deposited into the NCBI Sequence Read Archive (SRA) database by accession number PRJNA664217 (http://www.ncbi.nlm.nih.gov/bioproject/664217) and PRJNA664245 (http://www.ncbi.nlm.nih.gov/bioproject/664245).

## Supplementary Information

**Additional file 1 Table S1.** The abundance of core zokor gut bacterial communities in each group.

**Additional file 2 Table S2.** The most abundance OTUs in soil samples

**Additional file 3 Table S3.** The function of microbes of zokor and soil based on COGs

**Additional file 4 Fig. S1** Mean relative abundances of bacterial genera across zokors (LD, zokors from LD; PY, zokors from PY; YZ, zokors from YZ) and soil samples (LDI: soil inside the cave from LD; LDO: soil outside the cave from LD; PYI: soil inside the cave from PY; PYO: soil outside the cave from PY; YZI: soil inside the cave from YZ; YZO: soil outside the cave from YZ) at three different sites.

**Additional file 5 Fig. S2** Comparison of alpha diversity (Shannon and Chao index) of zokor and soil (outside the cave and inside the cave) bacterial communities in each site. (a) samples from LD. (b) samples from PY. (c) samples from YZ.

**Additional file 6 Fig. S3** Principal coordinates analysis (PCoA) of bacterial communities of zokors at three sites based on the Bray-Curtis distance metrics.

**Additional file 7 Fig. S4** Venn diagram showing the shared and unique OTUs among zokors at three sites.

**Additional file 8 Fig. S5** Relative abundance of shared OTUs between zokor samples and soil samples in each site. (a) samples from LD. (b) samples from PY. (c) samples from YZ.

**Additional file 9 Fig. S6** Pie charts showing the composition and relative abundance of shared OTUs between zokor samples and soil samples at phylum level in each site.

**Additional file 10 Fig. S7** Pie charts showing the composition and relative abundance of shared OTUs between zokor samples and soil samples at genus level in each site.

## Data Availability

The original sequence data during the current study are available at the SRA by accession number PRJNA664217 (http://www.ncbi.nlm.nih.gov/bioproject/664217) and PRJNA664245 (http://www.ncbi.nlm.nih.gov/bioproject/664245).
