# Supplementary figures and images for: The associations between intestinal bacteria of Eospalax cansus and soil bacteria of its habitat
Source: BMC Vet Res. 2022 Apr 2;18:129. doi: 10.1186/s12917-022-03223-6 (PMC8976338; doi:10.1186/s12917-022-03223-6)

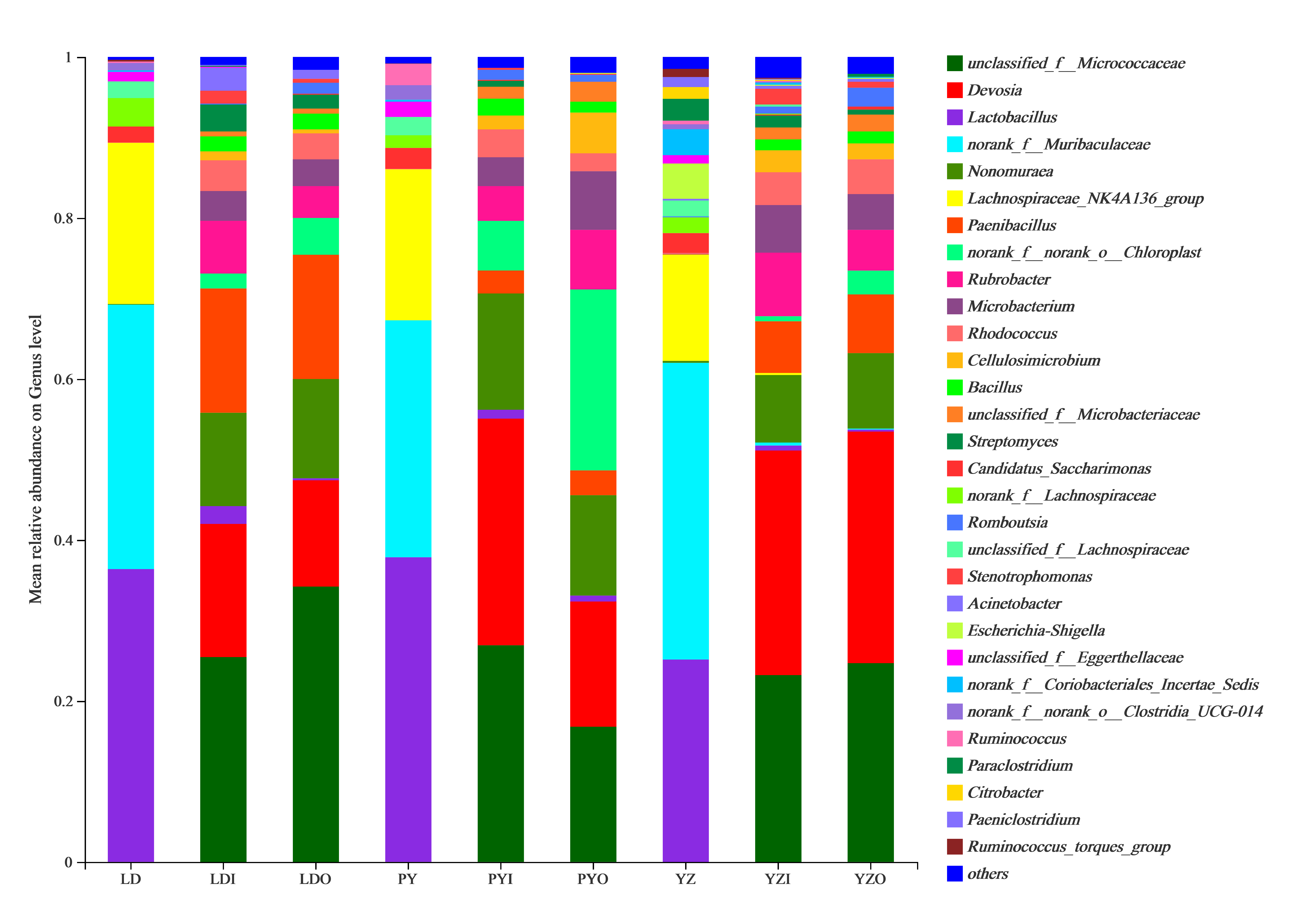

Supplement: Supplementary file 4 — Additional file 4 Fig. S1 Mean relative abundances of bacterial genera across zokors (LD, zokors from LD; PY, zokors from PY; YZ, zokors from YZ) and soil samples (LDI: soil inside the cave from LD; LDO: soil outside the cave from LD; PYI: soil inside the cave from PY; PYO: soil outside the cave from PY; YZI: soil inside the cave from YZ; YZO: soil outside the cave from YZ) at three different sites. [file 12917_2022_3223_MOESM4_ESM.png]

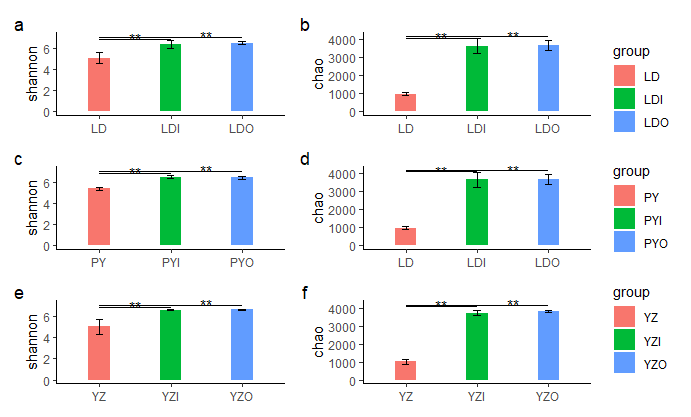

Supplement: Supplementary file 5 — Additional file 5 Fig. S2 Comparison of alpha diversity (Shannon and Chao index) of zokor and soil (outside the cave and inside the cave) bacterial communities in each site. (a) samples from LD. (b) samples from PY. (c) samples from YZ. [file 12917_2022_3223_MOESM5_ESM.png]

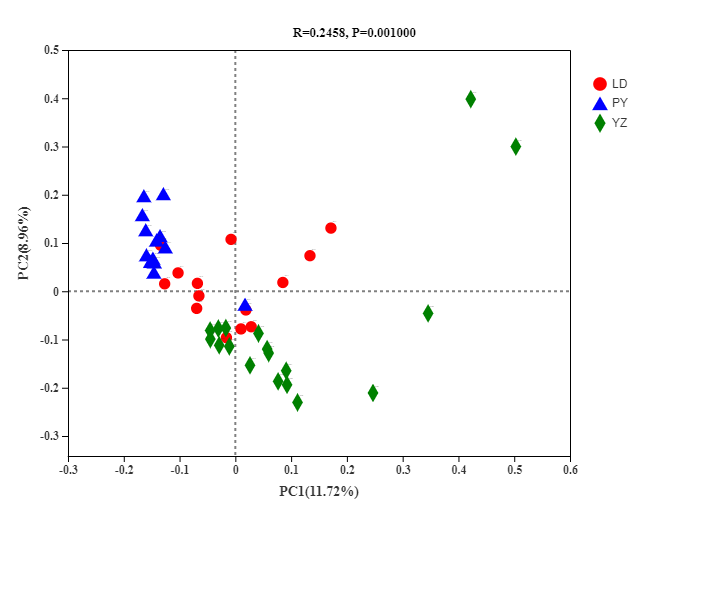

Supplement: Supplementary file 6 — Additional file 6 Fig. S3 Principal coordinates analysis (PCoA) of bacterial communities of zokors at three sites based on the Bray-Curtis distance metrics. [file 12917_2022_3223_MOESM6_ESM.png]

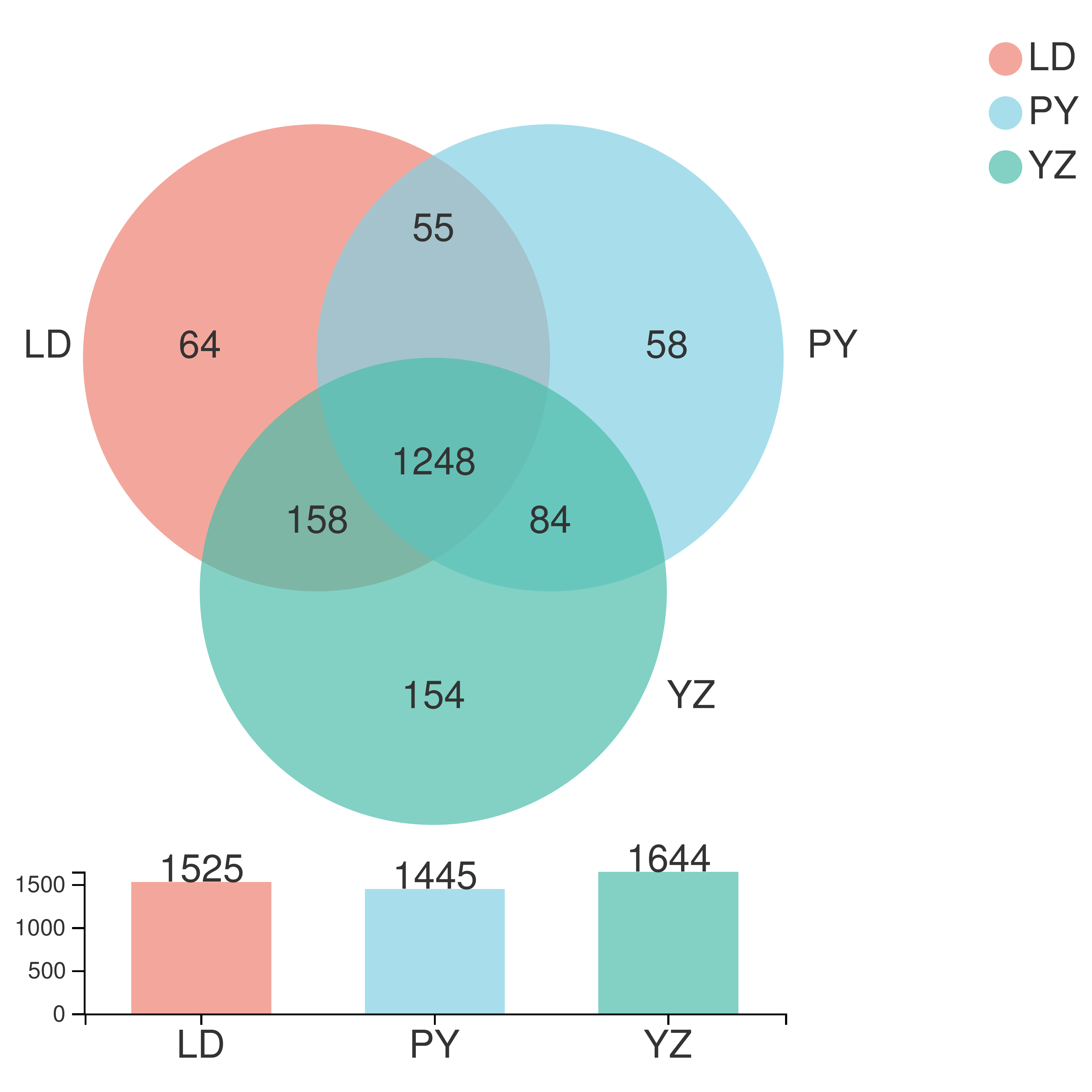

Supplement: Supplementary file 7 — Additional file 7 Fig. S4 Venn diagram showing the shared and unique OTUs among zokors at three sites. [file 12917_2022_3223_MOESM7_ESM.png]

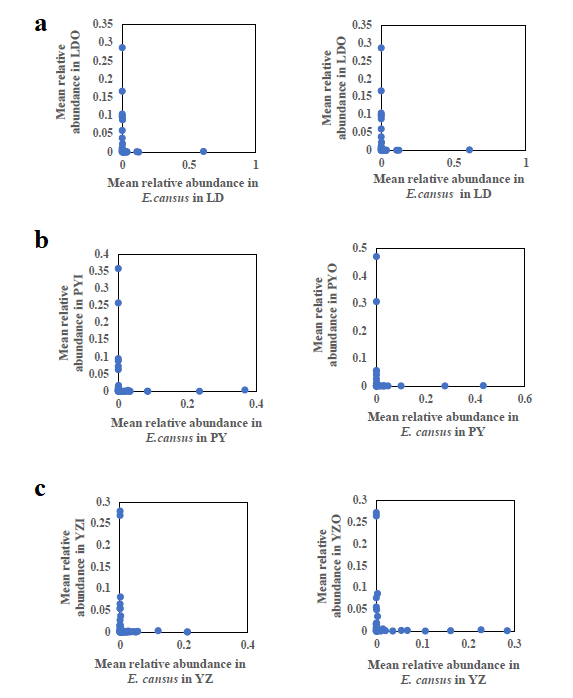

Supplement: Supplementary file 8 — Additional file 8 Fig. S5 Relative abundance of shared OTUs between zokor samples and soil samples in each site. (a) samples from LD. (b) samples from PY. (c) samples from YZ. [file 12917_2022_3223_MOESM8_ESM.png]

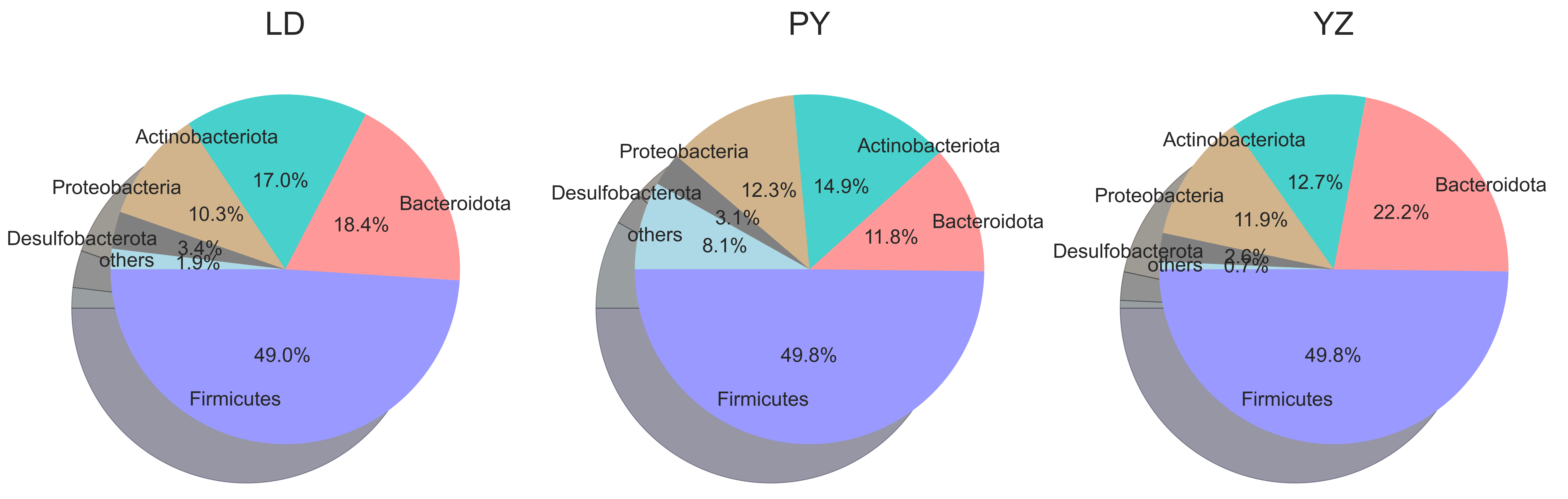

Supplement: Supplementary file 9 — Additional file 9 Fig. S6 Pie charts showing the composition and relative abundance of shared OTUs between zokor samples and soil samples at phylum level in each site. [file 12917_2022_3223_MOESM9_ESM.png]

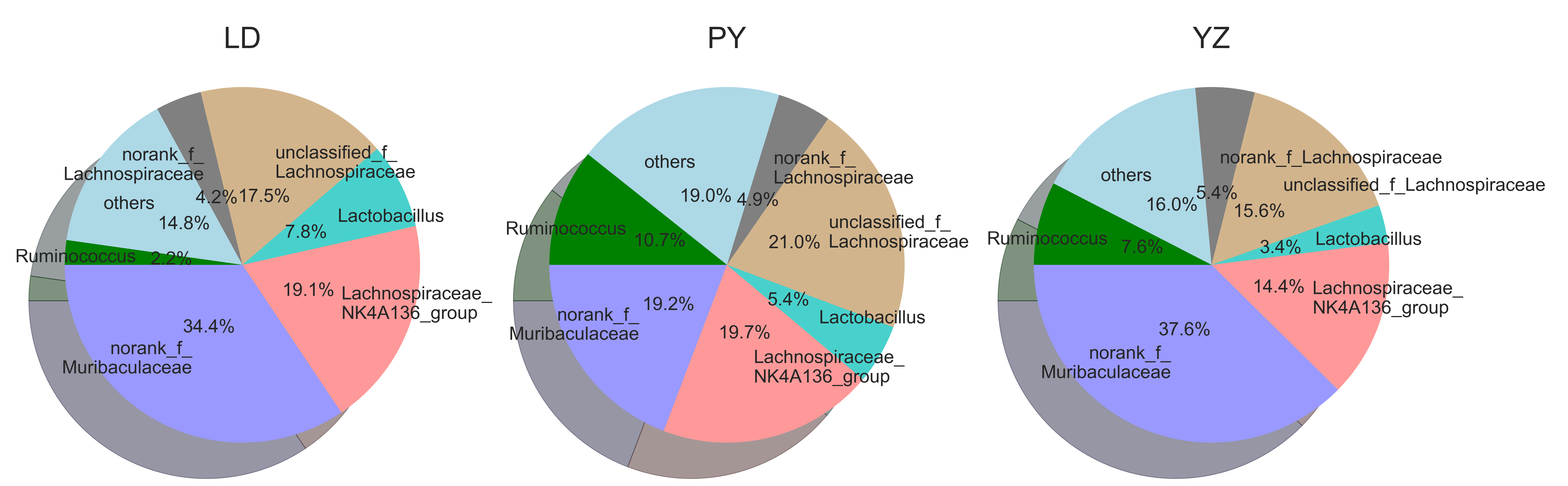

Supplement: Supplementary file 10 — Additional file 10 Fig. S7 Pie charts showing the composition and relative abundance of shared OTUs between zokor samples and soil samples at genus level in each site. [file 12917_2022_3223_MOESM10_ESM.png]
